# Adverse Events in Italian Nursing Homes During the COVID-19 Epidemic: A National Survey

**DOI:** 10.3389/fpsyt.2020.578465

**Published:** 2020-09-30

**Authors:** Flavia L. Lombardo, Emanuela Salvi, Eleonora Lacorte, Paola Piscopo, Flavia Mayer, Antonio Ancidoni, Giulia Remoli, Guido Bellomo, Gilda Losito, Fortunato D’Ancona, Marco Canevelli, Graziano Onder, Nicola Vanacore, The Italian National Institute of Health Nursing Home Study Group, Antonio Ancidoni

**Affiliations:** ^1^ National Center for Disease Prevention and Health Promotion, Italian National Institute of Health, Rome, Italy; ^2^ National Center for Drug Research and Evaluation, Italian National Institute of Health, Rome, Italy; ^3^ Department of Neuroscience, Italian National Institute of Health, Rome, Italy; ^4^ Department of Human Neuroscience, Sapienza University of Rome, Rome, Italy; ^5^ Unit of Health Care and Deprivation of Liberty, Rome, Italy; ^6^ Department of Infectious Diseases, Italian National Institute of Health, Rome, Italy; ^7^ Department of Cardiovascular, Endocrine-metabolic Diseases and Aging, Italian National Institute of Health, Rome, Italy

**Keywords:** dementia, adverse events, nursing homes, Long-Term Care Facilities, COVID-19, public health

## Abstract

Older people living in nursing homes (NHs) are particularly vulnerable in the ongoing COVID-19 pandemic, due to the high prevalence of chronic diseases and disabilities (e.g., dementia). The phenomenon of adverse events (AEs), intended as any harm or injury resulting from medical care or to the failure to provide care, has not yet been investigated in NHs during the pandemic. We performed a national survey on 3,292 NHs, either public or providing services both privately and within the national health system, out of the 3,417 NHs covering the whole Italian territory. An online questionnaire was addressed to the directors of each facility between March 24 and April 27, 2020. The list of NHs was provided by the Dementia Observatory, an online map of Italian services for people with dementia, which was one of the objectives of the implementation of the Italian National Dementia Plan. About 26% of residents in the Italian NHs for older people listed within the Dementia Observatory site had dementia. The objective of our study was to report the frequency of AEs that occurred during the months when SARS-CoV-2 spreading rate was at its highest in the Italian NHs and to identify which conditions and attributes were most associated with the occurrence of AEs by means of multivariate regression logistic analysis. Data are referred to 1,356 NHs that participated in the survey. The overall response rate was 41.2% over a time-period of six weeks (from March 24 to May 5). About one third of the facilities (444 out of 1,334) (33.3%) reported at least 1 adverse event, with a total of 2,000 events. Among the included NHs, having a bed capacity higher than the median of 60 beds (OR=1.57, CI95% 1.17–2.09; p=0.002), an observed increased in the use of psychiatric drugs (OR=1.80, CI95% 1.05–3.07; p=0.032), adopting physical restraint measures (OR=1.97, CI95% 1.47–2.64; p<0.001), residents hospitalized due to flu-like symptoms (OR =1.73, CI95% 1.28–2.32; p<0.001), and being located in specific geographic areas (OR=3.59, CI95% 1.81–7.08; OR = 2.90, CI95% 1.45–5.81 and OR = 4.02, CI05% 2.01–8.04 for, respectively, North-West, North-East and Centre vs South, p<0.001) were all factors positively associated to the occurrence of adverse events in the facility. Future recommendations for the management and care of residents in NHs during the COVID-19 pandemic should include specific statements for the most vulnerable populations, such as people with dementia.

## Introduction

During the COVID-19 pandemic, NHs in many countries were the among welfare settings most affected by the spread of the SARS-CoV-2 virus (1–4). NHs reported a high number of laboratory-confirmed cases of COVID-19 among residents, along with an increased number of residents deceased or hospitalized due to influenza-like symptoms (5–8). In particular, a national study including 9,395 NHs in the US, reported that 31.4% of the considered facilities had a documented case COVID-19 (9). A national survey involving 1,356 Italian NHs, reported that 29.0% of the facilities had at least one case of COVID-19 (10). At a regional level, in Ontario (Canada), 30.5% of NHs reported outbreaks of COVID-19 (11).

Case fatality rates among NHs residents ranged between 26% and 33.7% (5, 8, 9). In many countries, the number of deaths in NHs accounted for 21% to 50% of all the fatal cases of COVID-19 (12). In Italy, the cumulative incidence of hospitalizations of residents with laboratory-confirmed SARS-CoV-2 infection and with influenza-like symptoms was 1 and 2 per 100 residents, respectively (10).

Older people living in NHs are particularly vulnerable in the ongoing pandemic due to the high prevalence in this population of chronic diseases and disabilities (e.g., dementia). In particular, a systematic review of 74 studies examining the prevalence of psychiatric disorders and psychological symptoms in NHs, reported that a 58% median prevalence of dementia and a 78% prevalence of behavioural disorders among people with dementia (13). Accordingly, stricter guidelines have been defined for such vulnerable populations during the COVID-19 outbreak. Many National Health Authorities had recommended social distancing and limiting the access of visitors in NHs. As a result, older residents lost face-to-face contacts with their family members and caregivers, thus becoming socially isolated (14). These restrictive measures, necessary to avoid or limit the spreading of the infection, also resulted in an increase in behavioural disorders in patients with dementia during the pandemic (15). NHs actively found new means of communication to replace direct contacts, using videocalls and phone-calls. However, this means had a limited impact on residents with dementia, who need physical contact, a massage, and a nearby voice (16).

Hence, it can be assumed that the COVID-19 pandemic had an impact on NHs even beyond the extremely high number of deaths and hospitalizations. The epidemic and the measures adopted to contain its diffusion probably contributed to the occurrence of a wide range of adverse events (AEs) in long term care facilities, and specifically harms or injuries resulting from medical care, including the failure to provide needed care (17). These AEs may be classified in four groups: health care-acquired infections (i.e., catheter-associated urinary tract infection, respiratory infection); events related to residential care (i.e., falls causing injuries, pressure ulcers, confusion/delirium); events related to medication, (i.e., allergic reactions, delirium or other changes in mental status); events related to procedures (17).

The changing contingencies may have triggered negative events involving both residents and health care professionals, with potential important implications in terms of health outcomes, quality of life, and emotional status.

The objective of our study were: (i) to document the frequency of AEs that occurred in Italian NHs during the months in which the virus had its highest spreading rate (from start of February to the start of May); and (ii) to identify the determinants and attributes associated with the occurrence of AEs during the pandemic.

## Materials and Methods

This national survey involved 3,292 nursing homes, either public or providing services both privately and within the national health system, out of the 3,417 NHs covering the whole Italian territory. We included all the NHs for which we had an available reference contact. The list of NHs was provided by the Dementia Observatory, an online map of Italian services for people with dementia, which was one of the objectives of the implementation of the Italian National Dementia Plan (18, 19). In Italy, the majority of the NHs is located in the northern part of the country, the area that had the highest number of COVID-19 cases when the survey was carried out. In a previous study, we assessed prevalently the phenomenon of mortality and hospitalization during the COVID-19 pandemic in Italian NHs (10).

### Data Source

An online questionnaire with a cover letter was addressed to the directors of each NH between March 24 and April 27, 2020. NHs were subsequently also contacted by telephone to provide assistance in completing the questionnaire (about 3300 phone calls were made or received) Some of the NHs were further contacted to solve incongruences in some of the provided data. The 29 items of the online questionnaire were designed to gather information on: (1) the characteristics of the facility, including number of beds, type of structure (public or providing services both privately and within the NHS), number and type of healthcare and social workers (HCSW), residents living in the facilities; (2) the spreading of the infection, including the number of residents who died or were hospitalized due any cause occurred since February 1st, and those who were SARS-CoV-2 positive or had influenza-like symptoms, the number of hospitalizations within the considered time period, and the number of residents and staff members who had a SARS-CoV-2 positive test or influenza-like symptoms when the questionnaire was completed and the presence of positive cases among staff members; (3) the infection prevention and control (IPC) program components and practices for managing patients with suspected or confirmed COVID-19, including presence in the NH of written guidelines, availability of ad-hoc consultation, training for personnel, actions to raise awareness among residents (education of residents), ability to isolate patients, restriction of access for external visitors, and alternative means adopted to guarantee communication between residents and their relatives and caregivers (phone calls, video-calls), monitoring of possible early symptoms (temperature control twice daily), supply of alcohol-based hand sanitizers, and vaccination coverage for influenza. Moreover, the questionnaire included a question on potential difficulties faced during the pandemic; (4) issues potentially related to the epidemic, including use of physical restraint measures, increase in the use of psychoactive drugs (i.e., “Have you noticed an increase in the prescription of psychotropic medications -benzodiazepines, antidepressant or antipsychotic agents, since February 1?”), and AEs occurred since February 1st. (The full questionnaire is available in the **Supplementary Materials**). No information on individual residents and staff members were collected. On February 27, 2020, the Italian Presidency of the Council of Ministers authorized the collection and scientific dissemination of data concerning the COVID-19 epidemics by the INIH and other public health institutions.

### Definition of Adverse Events (AEs) and Physical Restraint

AEs were intended as any harm or injury resulting from medical care or to the failure to provide care. The directors of the surveyed NHs could report all the negative events occurring in their facilities involving residents and/or healthcare professionals. For instance, AEs could consist in falls, injuries, emotional suffering and behavioral disorders, delirium, adverse drug events, dehydration, bowel obstruction (17). Physical restraint was defined as “any action or procedure that prevents a person’s free body movement to a position of choice and/or normal access to his/her body by the use of any method, attached or adjacent to a person’s body that he/she cannot control or remove easily” (20).

### Statistical Analysis

We reported a description of the characteristics of the included NHs and of some aspects of their infection control and prevention (ICP) programs. We focused on data from the point 4 of the list reported above.

Descriptive statistics were performed on overall data and by region. Frequencies were used to describe discrete or dichotomous variables; mean and standard deviation (SD) were used for continuous variable, median, and range of values in case of asymmetric distribution. Missing data for the number residents were imputed using the number of beds. No other missing data were imputed. Univariate and multivariate regression logistic models were performed to assess whether some factors were associated to the occurrence of adverse events during the considered time period. We considered as study variables some characteristics of the included NHs (beds capacity, beds to staff ratio, geographical distribution), the difficulties faced during the pandemic, all information gathered on ICP, the spreading of COVID-19 in the NHs. The spreading of COVID-19 was assessed using the number of laboratory-confirmed cases among deceased and hospitalized residents, and among residents and/or staff members within the facility. The occurrence of death and hospitalization among residents with influenza-like symptoms was also investigated, to account for a possible underestimation of COVID-19 cases due to the potential lack of availability of swab tests in such a critical time, as, for example, the first period of the pandemic. All variables resulting as significant at 5% level in the univariate analysis were included in the multivariate model. Moreover, a sensitivity analysis was performed using the negative binomial regression model to assess the association between the number of events and the same considered factors. The exposure variable, i.e., the number of residents per facility, was included in the model.

All data analyses were performed using STATA software, version 14.2 (Stata Corp, College Station, Texas, USA).

## Results

This survey was addressed to both public structures and structures providing services both privately and within the NHS. However, 92 private NHs were also listed among the participating facilities and were included in the analyses.

Data are referred to 1,356 NHs that participated in the survey. The overall response rate was 41.2% over a time-period of 6 weeks (from March 24 to May 5). A negative association was observed between the response rate and the attack rate per region from the national surveillance system (20), even if not reaching statistical significance (Spearman’s rho= -0.21, p=0.344) (10). Two of the 21 regions (Valle D’Aosta and Basilicata) did not participate in the survey. At March, 24, the day the survey started, the regions where the spreading rate of COVID-19 was higher were Lombardy (303.6 per 100,000), Emilia Romagna (190.3), PA Trento (185.2 per 100,000) and Marche (175.7), while Basilicata was the region with the lowest attack rate (2.8 per 100,000 habitants) (21).

### Data Description and Spreading of the Infection in NHs

Overall, 100,806 persons were resident in the NHs participating to the survey (**Table 1**), with 77.2% of them located in the North of Italy. Overall, a mean of 74.7 beds (SD 57.6) per facility was reported, with a range between 8 and 667 beds and a median of 60 beds per facility. A huge variability was observed between Regions (**Table 1**). Considering the health care personnel operating in each facility, a median of 2 physicians, 7 nurses, and 24 health care social workers (HSCW) was reported per facility, with 11% of the NHs reporting that they had no physician within the facility. Overall, considering all the three professional figures, the staff included a median of 32 units (median value), corresponding to a mean of 1.8 beds (SD 1.1) per unit of staff.

**Table 1:** Distribution and description of facilities (number of NHs, residents, NHs with number of beds above the median, number of staff members, average number of beds per unit of staff (physicians, nurses, and health care social workers), by Region, and overall.

Italian Regions	NHsn (%)^a^	Residents^b^	NHs with beds above median^c^, N (%)	number of staff per facility^d^ median [range interquartile]	n. beds to staff ratio, mean ± sd
**Piedmont**	249 (41.0)	17186	130 (52.2)	26.5 [19–42.5]	2.3 ± 0.7
**Lombardy**	292 (43.1)	27657	222 (76.0)	42 [28–67]	2.2 ± 1.5
**AP Bolzano**	4 (10.8)	425	3 (100)	50 [26.5–127]	1.5 ± 0.5
**AP Trento**	15 (29.4)	1201	15 (100)	50 [46–68]	1.4 ± 0.2
**Veneto**	148 (28.5)	17902	122 (82.4)	57 [36–83]	1.9 ± 1.4
**Friuli V.G.**	39 (55.7)	3636	27 (69.2)	35.5 [25–53]	2.1 ± 0.7
**Liguria**	20 (17.2)	1573	11 (55.0)	25.5 [15–44]	2.4 ± 0.8
**Emilia Romagna**	128 (46.0)	8200	63 (49.2)	31 [24–45]	1.8 ± 0.7
**Tuscany**	200 (62.7)	9607	56 (28.0)	26 [17–36]	1.8 ± 1.2
**Umbria**	16 (38.1)	730	4 (25.0)	26 [13.5–38]	1.6 ± 0.2
**Marche**	36 (90.8)	1384	7 (19.4)	25 [18–29]	1.4 ± 0.4
**Latium**	79 (41.1)	4597	38 (48.1)	27.5 [17.5–42.5]	2.1 ± 0.7
**Abruzzo**	8 (49.0)	447	3 (37.5)	25 [19.5–35.5]	1.9 ± 0.7
**Molise**	4 (66.7)	233	2 (50.0)	26.5 [21–29]	2.6 ± 0.8
**Campania**	16 (13.2)	642	4 (25.0)	21.5 [17–27]	2.0 ± 0.5
**Apulia**	35 (57.4)	2088	18 (51.4)	26 [18–40]	2.0 ± 0.6
**Calabria**	36 (45.0)	1557	13 (37.1)	25.5 [16–39.5]	1.6 ± 0.4
**Sicily**	24 (61.5)	1132	7 (29.2)	25 [16.5–38]	1.5 ± 0.4
**Sardinia**	7 (43.8)	609	6 (85.7)	58 [37–67]	1.5 ± 0.3
**North-West**	561 (40.0)	46416	363 (64.7)	33 [22–54]	2.2 ± 1.2
**North-East**	334 (34.9)	31364	230 (69.1)	41.5 [28–68]	1.8 ± 1.1
**Centre**	331 (55.8)	16318	105 (31.7)	26 [17–36]	1.8 ± 1.0
**South and Islands**	130 (36.8)	6708	53 (41.1)	25 [18–39]	1.8 ± 0.6
**Overall**	**1356 (41.2)**	**100806**	**751(55.5)**	**32 [21**–**51]**	**1.8 ± 1.1**

AP, Autonomous Province.

^a^percentage on the total of NHs in the whole territory.

^b^Residents = people preset at 1^st^ February and new recovered since the 1^st^ March.

^c^median: 60 beds per facility.

^d^staff includes physicians, nurses and health care social workers.

North-West: Piedmont, Veneto, Liguria, Lombardy.

North-East: PA Bolzano, PA Trento, Veneto, Friuli Venezia Giulia, Emilia Romagna.

Centre: Tuscany, Umbria, Marche, Latium.

South and Islands: Abruzzo, Molise, Campania, Apulia, Basilicata, Calabria, Sicily, Sardinia.

Along with physicians, nurses, and HCSWs, the staff included also other healthcare professionals such as physiotherapists, psychologists, educators/animators, social workers, who were present respectively in 98.7%, 68.5%, 95.6%, and 45.3% of the NHs, reaching a median of 4 additional professionals (data not shown). Adverse events (AEs) were defined as any unfavorable event (e.g., accidents, confrontations, falls, aggressions) involving staff members, residents, or both staff members and residents. Information on the occurrence of AEs was based on the answers provided to the question if any AE occurred from February 1 to the date questionnaire was completed, and on the actual number of reported AEs, since some discrepancies were observed between the answers to these two questions. After checking for consistency, 14 NHs that answered “Yes” to the first question were recoded as “No” because they reported a number of 0 adverse events for all the types of AEs (involving residents, personnel, or both), while 6 NHs that answered “No” were recoded as “Yes” since they reported the number of events. Overall, one third of the facilities (444 out of 1,334) (33.3%) reported AEs, with a total of 2,000 events (**Table 2**). Most of the events involved only residents (92.1% of all events). An average of 2.1 events per 100 residents were reported, with a geographical trend showing higher values in the North-West area compared to the South and Islands (**Table 2**).

**Table 2:** Adverse events occurred between February 1st and the date the questionnaire was completed (between March 24 and May 5).

Italian Regions	NHs with adverse events, N (%)	Number of events	Events among personnel, N (%)	Events among residents, N (%)	Events involving staff and residents, N (%)	Cumulative incidence per 100 residents
**Piedmont**	76 (31.4)	428	9 (2.1)	404 (94.4)	15 (3.5)	2.6
**Lombardy**	117 (40.5)	621	11 (1.8)	590 (95.0)	20 (3.2)	2.3
**AP Bolzano**	0 (0.0)	0	0 (0.0)	0 (0.0)	0 (0.0)	0.0
**AP Trento**	11 (73.3)	85	0 (0.0)	82 (96.5)	3 (3.5)	8.9
**Veneto**	48 (33.1)	333	2 (0.6)	291 (87.4)	40 (12.0)	2.0
**Friuli V.G.**	14 (35.9)	54	0 (0.0)	47 (87.0)	7 (13.0)	1.6
**Liguria**	8 (40.0)	18	0 (0.0)	17 (94.4)	1 (5.6)	1.3
**Emilia Romagna**	41 (32.8)	122	4 (3.3)	111 (91.0)	7 (5.7)	1.6
**Tuscany**	68 (34.5)	176	8 (4.5)	151 (85.8)	117 (9.7)	1.9
**Umbria**	5 (31.3)	10	0 (0.0)	9 (90.0)	1 (10.0)	1.4
**Marche**	11 (30.6)	17	0 (0.0)	17 (100)	0 (0.0)	1.4
**Latium**	28 (36.3)	90	3 (3.3)	80 (88.9)	7 (7.8)	2.0
**Abruzzo**	1 (12.5)	1	0 (0.0)	1 (100)	0 (0.0)	0.2
**Molise**	0 (0.0)	0	0 (0.0)	0 (0.0)	0 (0.0)	0.0
**Campania**	0 (0.0)	0	0 (0.0)	0 (0.0)	0 (0.0)	0.0
**Apulia**	5 (14.3)	15	0 (0.0)	15 (100)	0 (0.0)	0.7
**Calabria**	4 (11.1)	11	0 (0.0)	11 (100)	0 (0.0)	0.7
**Sicily**	5 (20.8)	10	2 (20.0)	7 (70.0)	1 (10.0)	0.9
**Sardinia**	2 (28.6)	9	1 (11.1)	8 (88.9)	0 (0.0)	1.5
**North-West**	201 (36.5)	1067	20 (1.9)	1011 (94.8)	6 (3.4)	2.4
**North-East**	114 (34.8)	594	6 (1.0)	531 (89.4)	57 (9.6)	2.0
**Centre**	112 (34.6)	293	11 (3.8)	257 (87.7)	25 (8.5)	1.9
**South and Islands**	17 (13.2)	46	3 (6.5)	42 (91.3)	1 (2.2)	0.7
**Overall**	**444 (33.3)**	**2000**	**40 (2.0)**	**1841 (92.1)**	**119 (6.0)**	**2.1**

North-West: Piedmont, Veneto, Liguria, Lombardy.

North-East: PA Bolzano, PA Trento, Veneto, Friuli Venezia Giulia, Emilia Romagna.

Centre: Tuscany, Umbria, Marche, Latium.

South and Islands: Abruzzo, Molise, Campania, Apulia, Basilicata, Calabria, Sicily, Sardinia.

The physical restraint measures applied in the NHs during the period of investigation are reported in **Table 3**.

**Table 3:** Physical restraint measures by Region between February 1st and the date the questionnaire was completed (between March 24 and May 5).

Italian Regions	NHs adopting physical restraints, N (%)	Physical restraints, N	Median number [range interquartile]	Number of restraints per 100 residents
**Piedmont**	147 (64.2)	2260	2 [0–10]	15.3
**Lombardy**	190 (73.4)	4854	3 [0–21]	20.1
**PA Bolzano**	1 (25.0)	2	0 [0–1]	0.2
**PA Trento**	12 (80.0)	321	4 [2–37]	33.4
**Veneto**	84 (64.1)	3596	2 [0–30]	23.9
**Friuli Venezia Giulia**	19 (54.3)	342	1 [0–10]	12.0
**Liguria**	15 (78.9)	361	7 [1–27]	29.9
**Emilia Romagna**	80 (66.7)	1591	2 [0–15]	21.2
**Tuscany**	122 (65.6)	2056	3 [0–20]	25.1
**Umbria**	10 (66.7)	105	2 [0–9]	15.8
**Marche**	28 (84.8)	322	4 [1–17]	30.8
**Latium**	21 (27.3)	440	0 [0–1]	11.0
**Abruzzo**	2 (25.0)	30	0 [0–5]	7.7
**Molise**	2 (50.0)	6	1 [0–3]	2.4
**Campania**	3 (20.0)	40	0 [0–0]	8.4
**Apulia**	19 (59.4)	219	1.5 [0–6.5]	14.5
**Calabria**	5 (14.7)	17	0 [0–0]	1.2
**Sicily**	6 (28.6)	101	0 [0–1]	12.1
**Sardinia**	7 (100)	139	6 [2–26]	15.9
**North-West**	352 (69.4)	7475	3 [0–17]	18.3
**North-East**	196 (64.3)	5852	2 [0–20]	21.6
**Centre**	181 (58.2)	2923	2 [0–15]	21.8
**South and Islands**	44 (36.4)	552	0 [0–2]	8.8
**Overall**	**773(62.1)**	**16,802**	**2[0**–**15]**	**19.1**

In this survey, each resident may have received more than one measure of physical restraint. Up to 92.0% (1,221 over 1,327 NHs) of the facilities reported that they had a register and monitored all applied physical restraint measures. A total of 62.1% of the included NHs adopted physical restraints measures (773 out of 1,245). An average of 19 measures per 100 residents was reported, with a total number of 16,802 measures. A huge variability was observed across Regions (**Table 3**).

Overall, 29.0% of the NHs (387 out of 1,326) reported laboratory confirmed cases of COVID-19 among the deceased and/or hospitalized residents or among the staff members and residents living in the NHs when the questionnaire was completed. When considering also influenza like-symptoms the pandemic involved 909 out of the 1343 included NHs (67.7%) (10), with an overall cumulative incidence death rate of 9.1 per 100 residents (10). The rate of residents who died with laboratory confirmed COVID-19 was 0.7 per 100 residents, while the rate of residents who died with influenza-like symptoms was 3.1 per 100 residents (10).

Only 5.7% of the considered NHs reported an increase in the use of psychoactive drugs, mainly antipsychotics and benzodiazepine.

### Description of Data on IPC Programs Applied in the NHs

When considering aspects of IPC procedures, written guidelines for the appropriate management of residents with COVID-19 were available in 92.9% of the NHs, but 59.4% of the facilities did not receive any ad-hoc consultation for neither the management of patients nor for IPC. No specific training for COVID-19 was provided to staff members in 35.1% of the NHs, while 93.3% of the NHs provided some training on the appropriate use of personal protective equipment (PPE). Moreover, 91.5% of the NHs provided information and raised awareness on COVID-19 among residents.

All the NHs, except for one, suspended all visits from relatives/caregivers to the residents (in agreement with the legislation issued on March 8, 2020), with almost all of them (99.5%) providing alternative means for communication. The most frequently adopted alternative were videocalls (89.6%).

As for the main difficulties faced during the pandemic, 77.2% of the 1259 NHs that answered this question reported a lack of PPE, 52.1% were not able to obtain laboratory tests (data available starting April, 9, thus referring to 541 NHs), 33.8% reported lack of personnel, 26.2% had difficulties in isolating patients with COVID-19, and 12.5% had difficulties in transferring patients to hospitals. A total of 20.9% of the NHs reported they received scarce information on the procedures to be carried out to contain the infection, and 9.8% reported a lack of drugs.

Up to 7.7% of the NHs were not able to isolate residents with suspected or confirmed COVID-19. An in-depth analysis showed that facilities with a lower bed capacity had a higher probability of not being able to isolate residents with confirmed or suspected COVID-19. Specifically, 4.5% of the facilities with more than 60 beds (n=747) were not able to isolate residents compared to 11.6% of NHs with less than 60 beds.

Almost all the NHs (99.9%) provided alcohol-based hand sanitizers to their staff members. Most of the included NHs (79.1%) reported to monitor the temperature among residents and staff members twice a day.

A total of 1045 NHs reported data on influenza vaccination, with an overall median coverage of 95%. Only 16.2% of the NHs reported a coverage lower than 75%.

### Association With Adverse Events

As AEs were more frequent in North Italy, the area where the highest number of NHs with high bed capacity were located and where the spreading of COVID-19 was higher, these aspects were further explored to assess their potential association with the occurrence of AEs. Moreover, the beds to staff ratio was also investigated, as a potential proxy of the quality of assistance. The association between AEs and spreading of COVID-19 in the facility was also evaluated, considering the presence of cases of COVID-19 reported among residents (deceased, hospitalized or still living in the facility) and staff members. Furthermore, due to the lack of availability of swab tests in some contexts, in particular during the early phases of the pandemic, information about deceased and hospitalized residents with influenza-like symptoms were also considered. Potential associations with use of physical restraints, difficulties during the pandemic, aspects of IPC, use of alternative means of communication with relatives/caregivers (videocalls, phone calls or other means), and increased use of psychoactive drugs were also explored. In particular, the purpose of this last question was to understand whether there had been an increase in the prescriptions of these categories of drugs from February 1.

Univariate and multivariate logistic models were performed in order to investigate the association among these aspects and the likelihood of occurrence of AEs.

The multivariate analysis showed that facilities with beds capacity higher than the median of 60 beds (OR=1.57,CI95% 1.17–2.09; p=0.002), an observed increase in the use of psychoactive drugs (OR=1.80, CI95% 1.05–3.07; p=0.032), the adoption of physical restraint measures (OR=1.97, CI95% 1.47–2.64;p<0.001), the occurrence hospitalizations of residents with flu-like symptoms (1.73, CI95% 1.28–2.32; p<0.001) and the geographic area (OR=3.59, CI95% 1.81–7.08 for North-West, OR = 2.90, CI95% 1.45–5.81 for North-East and OR = 4.02, CI 95% 2.01–8.04 for Centre vs South, p<0.001) were all positively associated with the occurrence of AEs in the facility. Lack of personnel, difficulties in isolating patients, spreading of COVID-19 within the facility, and presence of residents deceased with influenza-like symptoms, all lost statistical significance in the multivariate models (**Table 4**). The sensitivity analysis conducted on the number of AEs performing the negative binomial model, despite the lack of convergence for some factors, confirmed which factors were associated with AEs and no further factors were identified. All the other variables of ICP not included in **Table 4** resulted non statistically significant in the comparison between NHs and AEs and those not at the univariate analysis.

**Table 4:** Crude and adjusted ORs by univariate and multivariate logistic model, estimating the association with the occurrence of adverse events in nursing home (NHs).

Variables	Crude OR			Adjusted OR^a^		
	OR_cr_	p-value	95%CI	OR_adj_^a^	p-value	95%CI
**Lack of personnel** (Y vs N)	1.38	0.010	1.08–1.77	0.96	0.786	0.71–1.29
**Difficulty in isolating** (Y vs N)	1.42	0.008	1.09–1.85	1.21	0.227	0.89–1.63
**Number of beds** (upper vs below the median*)	1.74	<0.001	1.38–2.21	1.57	0.002	1.17–2.09
**Increased use of psychoactive drugs** (Y vs N)	2.09	0.002	1.31–3.32	1.80	0.032	1.05–3.07
**Physical restraints** (Y vs N)	2.37	<0.001	1.83–3.08	1.97	<0.001	1.47–2.64
**COVID-19 spreading** (Y vs N)	1.57	<0.001	1.22–2.01	1.08	0.663	0.77–1.50
**Deaths with influenza-like symptoms** (Y vs N)	1.66	<0.001	1.32–2.09	1.00	0.990	0.73–1.36
**Hospitalization with influenza-like symptoms** (Y vs N)	2.10	<0.001	1.66–2.65	1.73	<0.001	1.28–2.32
**Geographic Region** (vs South)						
North-West	3.78	<0.001	2.21–6.48	3.59	<0.001	1.81–7.08
North-East	3.51	<0.001	2.01–6.14	2.90	0.003	1.45–5.81
Centre	3.45	<0.001	1.97–6.03	4.02	<0.001	2.01–8.04

^a^Adjusted for all the variables listed in the table.

*the median of beds per facility was 60 beds.

## Discussion

In this study, we observed that one third of the included facilities (33.3%) reported at least one AE, with a total of 2,000 events. Most of the events involved only residents. A geographical trend was observed, with higher values in the North-West area compared to the South and Islands. Overall, 29.0% of the NHs reported at least one laboratory-confirmed case of COVID-19 among residents and staff members. When considering also influenza-like symptoms 67.7% of NHs reported at least one case.

The NHs that reported AEs also reported a higher frequency of use of psychoactive drugs and physical restraint when compared to those that did not report any AEs. The strong association between these variables is likely a reflection of a critical context in the daily management of residents. An association between AEs and NHs with a higher number of beds and those with a higher number of residents hospitalized due to flu-like symptoms was also reported. All these variables contributed to define a pattern of the facilities who faced critical situations during the pandemic.

Our study showed that the NHs located in Central Italy and Northern Italy had a higher risk of AEs compared to those in the South-Islands. This trend is not in line with the spread of COVID-19 in Italy, as the most affected regions were those in the Northern area.

This may mean that probably the specific care setting of the NHs, with a large number of residents and part of them requiring an hospitalization, had a higher weight in determining the frequency of conflicts rather than the spreading of COVID-19 in general population.

A study conducted in 2015–2019 on a sample of 330 Italian NHs for older people taken from the Dementia Observatory register, showed that about 26% of residents had dementia (22). A similar frequency was also reported by a systematic review including studies focusing specifically on NHs (13).

Residents with dementia in NHs need a higher level of care than other residents due to a higher number of both non-self-sufficient individuals and people with behavioral disorders. During quarantine, the usual care routine was radically changed and several facilities probably reduced, if not suspended, the set of non-pharmacological treatments. This, along with the suspension of visits from relatives/caregiver, who are often able to calm residents with cognitive impairment, can result in an onset or an increase of behavioral symptoms (23).

The use of technologies such as videocalls or/and calls is likely to be less effective in residents with dementia than in other types of residents (16, 24). However, in our study this variable did not show any association with a lower probability of AEs in NHs.

In our study we did not analyze the different components of adverse events (falls, injuries, emotional suffering and behavioral disorders, delirium, adverse drug events, dehydration, bowel obstruction) and thus were not able to perform any comparison with the pre-pandemic situation.

There are few data published on the frequency of specific AEs in Italian NHs (25–27). However, potentially inappropriate drug prescriptions, an increased risk of falls causing injuries in residents with cognitive impairment, and an high prevalence of behavioral disorders in people with dementia were reported in NHs (13, 25, 28). Overall, it is known that mistakes in medications, falls, delayed or inappropriate interventions, and lacking or inadequate care contribute to AEs. The most commonly identified contributing factors were lack of expertise, lacking or incomplete documentation, failure to work as a team, and inadequate communication (29). These factors may have been more common during the pandemic. Moreover, nurses in NHs can play a relevant role in reporting and reducing adverse events, and a routine monitoring should be considered as a quality and safety indicator (30, 31).

The main strength of our study is reporting the results of a national survey carried out during the most critical phase of the pandemic.

### Limitations

Limits are mainly due to a lack of data on persons with dementia and a 41% response rate. In particular, to our knowledge, this is the first study that reports living conditions within a large number of NHs included in the Italian Dementia Observatory in which about 26% of residents had a diagnosis of dementia (22).

In an attempt to characterize the non-response bias in our survey, we observed an inverse correlation between the response rate to the survey across the different regions and the corresponding infection attack rate per region. Moreover, we identified about 53 NHs from news report that did not respond to the survey and had outbreaks of COVID-19 with a consistent number of deaths and a high frequency of people hospitalized due to flu-like symptoms.

We believe that NHs that had problems during the pandemic might have not responded to our survey, and thus the results of this study might probably underestimate AEs and other variables that characterize the level of assistance in a NHs.

We are also aware that the answers provided to our questionnaire may not be accurate (i.e., different understanding of physical restraints, psychotropic medication or AEs by the respondents) nor true, and a validity study was not performed (the administration of a second questionnaire in a sub-sample of NHs). In particular, we could not check whether all respondents to the questionnaire considered all the events included in the definition of adverse events used in literature. We also underlined that for some questions the quantitative data on the pre-COVID-19 period were not available (i.e., the use of psycho-active drugs, adverse events, physical restraint measures). Therefore so, we could not be able to perform any comparisons with the data collected with the survey. Moreover, the wide variability of these data could be explained by a different level of participation in answering to these specific questions.

Future recommendations issued by governmental agencies, scientific societies and public institutions for the management and care of residents in NHs during the COVID-19 pandemic should include specific statements for the most vulnerable populations, such as people with dementia (12, 32–36).

## Data Availability Statement

The raw data supporting the conclusions of this article will be made available by the authors, without undue reservation.

## Ethics Statement

On February 27, 2020, the Italian Presidency of the Council of Ministers authorized the collection and scientific dissemination of data concerning the COVID-19 epidemics by the INIH and other public health institutions. Therefore, ethical review and approval was not required for the study on human participants in accordance with the local legislation and institutional requirements.

## List of the Italian National Institute of Health Nursing Home Study Group co-authors

Antonio Ancidoni, Ilaria Bacigalupo, Guido Bellomo, Luigi Bertinato, Marco Canevelli, Patrizia Carbonari, Maria Grazia Carella, Annamaria Confaloni, Alessio Crestini, Fortunato D’Ancona, Carla Faralli, Simone Fiaccavento, Silvia Francisci, Flavia L. Lombardo, Eleonora Lacorte, Cinzia Lo Noce, Paola Luzi, Tania Lopez, Flavia Mayer, Maria Masocco, Monica Mazzola, Graziano Onder, Ilaria Palazzesi, Luana Penna, Daniela Pierannunzio, Paola Piscopo, Maria Cristina Porrello, Giulia Remoli, Emanuela Salvi, Giulia Scaravelli, Andrea Siddu, Sabrina Sipone, Lucia Speziale, Andrea Tavilla, Nicola Vanacore (Italian National Institute of Health).

Mauro Palma (President) and Gilda Losito (Head of Unit - Deprivation of liberty and Health protection), Italian National Guarantor for the rights of persons detained or deprived of liberty

Gianluca Pucciarelli (Dipartimento di Biomedicina e Prevenzione-Università di Tor Vergata), Daniela Accorgi (UsL Centro Toscana), Catia Bedosti (Ausl Imola- Emilia Romagna), Gabriella Carraro (Aulss 2 Veneto) Maria Mongardi (Dipartimento di Malattie Infettive – Università di Verona)

Gianluca Ferrari (Area Comunicazione e Informatica srl).

## Author Contributions

NV, GO, FD’A, MC, and GL conceived the study. FL and FM worked on statistical aspects of the study. IB, ES, EL, PP, AA, GR, and GB were involved in the organization of the study. The Italian National Institute of Health Nursing Home Study Group performed the study. All authors contributed to the article and approved the submitted version.

## Conflict of Interest

The authors declare that the research was conducted in the absence of any commercial or financial relationships that could be construed as a potential conflict of interest.
