# Supplementary material for: Adverse Events in Italian Nursing Homes During the COVID-19 Epidemic: A National Survey
Source: Front Psychiatry. 2020 Sep 30;11:578465. doi: 10.3389/fpsyt.2020.578465 (PMC7561357; doi:10.3389/fpsyt.2020.578465)
Supplement: Supplementary file 1 [file Table_1.docx]

**English version of the Questionnaire**

**
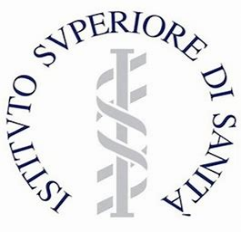
**

**QUESTIONNAIRE ON COVID-19 INFECTION IN NURSING HOMES**

**Name of the structure**

**Type of structure:**

**options:** Providing services within the NHS [ ]

Providing services both privately and within the NHS [ ]

Providing services only privately [ ]

*Other* [specify: ]

**Business name:**

**Address:**

**City:**

**Province/District:**

**Region:**

**Phone number (you can provide more than one number):**

**E-mail address:**

**Who is completing the questionnaire:**

**First name and last name:**

**Role within the structure:**

**E-mail address:**

**Data in which the questionnaire was completed:**

**1. What is the total number of healthcare and social workers (HCSW) who are currently operating in the structure?**

- Physicians, specify the total number: [ ]

- Nurses, specify the total number: [ ]

- Other HCSW, specify the total number: [ ]

*other members of the staff that could have been in close contact with the patients (e.g. educators, entertainers, psychologists, etc.):*

*-* Social workers, number: [ ]

*-* Physiotherapists/therapists/rehabilitators, number: [ ]

- Psychologists, number: [ ]

- Educators/entertainers, number: [ ]

- Other, specify type: number: [ ]

**2. How many beds are available in the facility?**

Number:  [ ]

**3. How many residents were in the facility on February 1?**

Number:  [ ]

**4. How many residents have died since February 1?** [ ]

Specify the number for each time period:

**February 1-15:** [ ]

**February 16-29:** [ ]

**March 1-15:** [ ]

**March 16-31:** [ ]

**April 1-15:** [ ]

**April 16 -30:** [ ]

**5. How many residents who died in the facility since February 1 had a positive swab test for COVID-19?**

Number:  [ ]

**6. Overall, how many residents who died in the facility since February 1 had influenza-like symptoms, respiratory symptoms (such as fever, chough or dyspnea) or pneumonia (irrespective of their having undergone a test for COVID-19)?**

 Number:  [ ]

**7. How many residents have been hospitalised since February 1?**

Number:  [ ]

**8. How many residents who were hospitalised since February 1 had a positive swab test for COVID-19?**

  Number:  [ ]

**9.** **How many residents who were hospitalised since February 1 had influenza-like symptoms, respiratory symptoms (such as fever, chough or dyspnea) or pneumonia (irrespective of their having undergone a test for COVID-19)?**

Number: [ ]

**10. How many residents were newly admitted to your facility since March 1?**

Number: [ ]

**11. How many patients are currently present in your facility who either have a positive swab test for COVID-19 or influenza-like symptoms/pneumonia?**

- positive swab test for COVID-19, number: [ ]

- influenza-like symptoms/pneumonia, (irrespective of having undergone a test for COVID-19, number: [ ]

**12. What are the main difficulties you are facing during the coronavirus epidemics?**

(more that one option is allowed, mark with an X)

a.     Lack of information provided on the procedures to be carried out to contain the infection [ ]

b.    Shortage of drugs and medications

c.     Lack of personal protective equipment (PPE) [ ]

d.    Absence of personnel [ ]

e.     Difficulty in transferring the residents with COVID-19 to hospitals [ ]

f.      Difficulties in isolating the residents with COVID-19 [ ]

g. Impossibility of having suspect cases undergo a swab test for Sars-Cov-2

f.     Other (specify):

**13.  In agreement with the legislation (DPCM) issued March 8, 2020, did you suspend all visits from relatives/caregivers to the residents?**

- No
- If yes, specify since when the visits were suspended: mm/dd/yyyy
- If visits were allowed, specify any exceptions (e.g. end of life, other):

**14. Did you provide alternative means for communication between residents and their relatives/caregivers?**

- NO [ ]

- YES [ ]

If yes, specify:

- since when the visits were suspended: mm/dd/yyyy

- which means were provided:

- videocalls No [ ] Yes[ ]

- phone calls No [ ] Yes [ ]

- other (specify):

**15. Did any member of the staff in the facility have a positive swab test for COVID-19?**

- NO [ ]

- YES [ ]

**16. Do you have a written plan/procedure for the management of residents with suspected or confirmed COVID-19?**

- NO [ ]

- YES [ ]

**17. Did you receive an ad hoc consultation for the clinical management and/or prevention and control of COVID-19?**

- NO [ ]
- YES [ ] (specify the type of consultation):

**18. Who is in charge of managing the residents with suspected or confirmed COVID-19?**

 (mark with an X)

a.     GPs [ ]

b.    Physicians within the facility [ ]

c.     External consultants [ ]

d.    Other (specify): [ ]

**19. Are you able to isolate residents in case of suspected or confirmed Sars-Cov-2 infection?**

 (mark with an X)

- No[ ]
- Yes (private room) [ ]
- Yes (room with only residents with COVID-19) [ ]
- Yes (transferal to another facility) [ ]
- Yes (other specify): [ ]

**20. Does the facility register and monitor all applied physical restraint measures?**

- NO [ ]
- YES [ ]

**21. How many physical restraint measures were applied since February 1 for the management of the residents?**

- Number: [ ]

**22. Have you noticed an increase in the prescription of psychotropic medications (benzodiazepines, antidepressant or antipsychotic agents) since February 1?**

- NO [ ]

- YES [ ]

If YES, specify for what type of medication:

- benzodiazepines [ ]

- antidepressant drugs [ ]

- antipsychotic drugs [ ]

**23. Have you noticed any adverse event (e.g. accidents, confrontations, falls…) since February 1?**

- NO [ ]

- YES [ ]

If yes, specify the number of:

- Adverse events among the staff: [ n° ]
- Adverse events among the residents: [ n° ]
- Adverse events involving both the staff and residents: [ n° ]

**24. Have you implemented a training program for healthcare and social workers including also specific practical activities on COVID-19 (NIH courses, videos…)?**

- NO[ ]
- YES [ ]

**25*.* Have you implemented a training course for healthcare and social workers on the appropriate use of PPE?**

- NO [ ]
- YES [ ]

**26. Did you take any initiative to share information and raise awareness among residents on the prevention and control of COVID-19?**

- NO [ ]
- YES [ ]

**27. Are there hand sanitizer dispensers available for staff to use?**

- NO [ ]
- YES [ ]

**28. Do you measure the temperature twice a day among both residents and staff members?**

- NO [ ]
- YES [ ]

**29. What is the influenza vaccine coverage of the residents in the facility?**

- Specify the percentage: [ ]

**Thank you for your cooperation**
